# Liver injury in non-alcoholic fatty liver disease is associated with urea cycle enzyme dysregulation

**DOI:** 10.1038/s41598-022-06614-9

**Published:** 2022-03-01

**Authors:** Rocío Gallego-Durán, Javier Ampuero, Helena Pastor-Ramírez, Leticia Álvarez-Amor, Jose Antonio del Campo, Douglas Maya-Miles, Rocío Montero-Vallejo, Antonio Cárdenas-García, Mª Jesús Pareja, Sheila Gato-Zambrano, Raquel Millán, María del Carmen Rico, Amparo Luque-Sierra, Antonio Gil-Gómez, Ángela Rojas, Rocío Muñoz-Hernández, María García-Lozano, Rocío Aller, Raúl J. Andrade, Carmelo García-Monzón, Fausto Andreola, Francisco Martín, Rajiv Jalan, Manuel Romero-Gómez

**Affiliations:** 1 SeLiver Group, Instituto de Biomedicina de Sevilla/Hospital Universitario Virgen del Rocío/CSIC/Universidad de Sevilla, Avda. Manuel Siurot s/n, 41013 Sevilla, Spain; 2 Hepatic and Digestive Diseases Networking Biomedical Research Centre (CIBERehd), Madrid, Spain; 3 UCM Digestive Diseases Unit, Hospital Universitario Virgen del Rocío, Avda. Manuel Siurot s/n, 41013 Sevilla, Spain; 4 Andalusian Center of Molecular Biology and Regenerative Medicine-CABIMER, University Pablo Olavide-University of Seville-CSIC, Sevilla, Spain; 5 Biomedical Research Network On Diabetes and Related Metabolic Diseases-CIBERDEM, Instituto de Salud Carlos III, Madrid, Spain; 6 Digestive Diseases Unit, Hospital Virgen de Valme, Sevilla, Spain; 7 Pathology Unit, Hospital Virgen de Valme, Sevilla, Spain; 8 Digestive Diseases Unit, Hospital de Valladolid, Valladolid, Spain; 9 Unit for the Clinical Management Gastroenterology, Instituto de Investigación Biomédica de Málaga-IBIMA, Hospital Universitario Virgen de La Victoria, Universidad de Málaga, Málaga, Spain; 10 Liver Research Unit, Hospital Universitario Santa Cristina, Instituto de Investigación Sanitaria Princesa, Madrid, Spain; 11 Liver Failure Group, Institute for Liver and Digestive Health, Royal Free Hospital, London, UK

**Keywords:** Gastroenterology, Health care, Medical research

## Abstract

The main aim was to evaluate changes in urea cycle enzymes in NAFLD patients and in two preclinical animal models mimicking this entity. Seventeen liver specimens from NAFLD patients were included for immunohistochemistry and gene expression analyses. Three-hundred-and-eighty-two biopsy-proven NAFLD patients were genotyped for rs1047891, a functional variant located in carbamoyl phosphate synthetase-1 (CPS1) gene. Two preclinical models were employed to analyse CPS1 by immunohistochemistry, a choline deficient high-fat diet model (CDA-HFD) and a high fat diet LDLr knockout model (LDLr −/−). A significant downregulation in mRNA was observed in CPS1 and ornithine transcarbamylase (OTC1) in simple steatosis and NASH-fibrosis patients *versus* controls. Further, age, obesity (BMI > 30 kg/m^2^), diabetes mellitus and ALT were found to be risk factors whereas A-allele from CPS1 was a protective factor from liver fibrosis. CPS1 hepatic expression was diminished in parallel with the increase of fibrosis, and its levels reverted up to normality after changing diet in CDA-HFD mice. In conclusion, liver fibrosis and steatosis were associated with a reduction in both gene and protein expression patterns of mitochondrial urea cycle enzymes. A-allele from a variant on CPS1 may protect from fibrosis development. CPS1 expression is restored in a preclinical model when the main trigger of the liver damage disappears.

## Introduction

Non-alcoholic fatty liver disease (NAFLD) is currently considered the hepatic manifestation of the metabolic syndrome^1^. Non-alcoholic steatohepatitis (NASH) is a condition histologically characterized by inflammation, ballooning degeneration and may be or not accompanied by liver fibrosis, together with the typical fatty hepatocytes, in absence of relevant alcohol consumption. This entity has surfaced as a major health problem worldwide, due to its high global prevalence, reaching 25%, and not negligible hepatocellular carcinoma progression and mortality rates^2^. Liver fibrosis is the strongest predictor of mortality in NAFLD patients^3^, and it has been recently reported that significant fibrosis forecasts new onset *diabetes mellitus* and arterial hypertension in NASH patients^4^. The global outbreak of obesity fuels metabolic disarrangements and predicts a higher socioeconomic burden of this disease^2^.

Nitrogen is essential for life-maintenance, and urea cycle is critical for the clearance of the excess of nitrogen that otherwise could be primarily accumulated as ammonia. Thus, urea cycle enzymes (UCEs) have been matter of study for a long time. Previous studies in different NAFLD animal models and clinical settings have demonstrated that messenger RNA from UCEs seems to be compromised, even resulting in a functional reduction in ureagenesis capacity^5^. Further, a recent study has shown that inhibition of urea-cycle flux generating carbamoyl phosphate synthetase 1 (CPS1) correlated with a loss of functionality for ureagenesis in NASH, whereas glutamine synthetase (GS) was increased^6^. Indeed, the use of ammonia scavenger ornithine phenylacetate prevented from hepatocyte death and diminished in vitro and in vivo liver fibrosis development^7^. NASH might produce a reversible reduction in UCEs expression and function, resulting in an impaired ammonia clearance and might generate hyperammonemia through the activation of hepatic stellate cells^8^. Moreover, rs1047891 variant located in CPS1 gene has been previously associated with functional consequences of the downstream availability of L-ornithine, precursor of nitric acid^9^. Therefore, it has been linked to several clinical conditions, such as differential serum lipid profiles in certain ethnic groups^10^ and A-allele carriers seem to be more prone to develop hyperammonemia associated to valproic acid intake for the treatment of epilepsy^11^.

The lack of effective therapies to treat NAFLD and the high rate failure in randomized clinical trials, lead us to think that there is still unravelled pathogenic mechanisms, such as ammonia, that could influence disease progression. In this scenario, considering that NAFLD molecular mechanisms are not properly unravelled yet, there is a shortage of effective therapeutic treatments apart from lifestyle interventions^12^. Therefore, the aim of this study was to evaluate changes in the urea cycle enzymes in patients with NAFLD and in two preclinical animal models mimicking this disease.

## Results

### UCEs gene and protein expression results

Snap frozen and paraffin-embedded liver tissue was available for both gene expression and immunohistochemistry (IHC) purposes from 17 NAFLD biopsy-proven patients. To avoid biases, patients were selected as bland steatosis (n = 10), defined as steatosis presence, nor steatohepatitis or fibrosis, and NASH with fibrosis (n = 7). Seven healthy controls were also included for gene expression analyses. Main features of the cohort included in both IHC and gene expression analyses are shown in Table 1. Briefly, mean age was 44.1 ± 13.1 years, 47% (8/17) were male, and 41.2% (7/17) displayed significant fibrosis in liver biopsy.

**Table 1:** Clinical and analytical characteristics of the study cohort employed for immunohistochemistry and gene expression analyses.

Variable	Overall cohort (n = 17)
Age, years	44.1 ± 13.1
Sex distribution (male), %	47.1% (8/17)
BMI (kg/m^2^)	29.04 ± 5.02
T2DM, %	29.4% (5/17)
Arterial hypertension, %	29.4% (5/17)
Dyslipidemia, %	52.9% (9/17)
AST (IU/mL)	41 ± 23
ALT (IU/mL)	55 ± 34
GGT (IU/mL)	95 ± 105
Glucose (mg/dL)	100 ± 41
Insulin (microUI/mL)	13 ± 9
Triglycerides (mg/dL)	119 ± 87
Total cholesterol (mg/dL)	190 ± 46
Albumin (mg/dL)	4252 ± 310
Platelet count, ^10^9^	207 ± 56
Significant fibrosis (F2–F4), %	41.2% (7/17)

*BMI* body mass index, *T2DM* type 2 diabetes mellitus, *AST* aspartate aminotransferase, *ALT* alanine aminotransferase, *GGT* gamma glutamil transferase.

**Figure 1:**
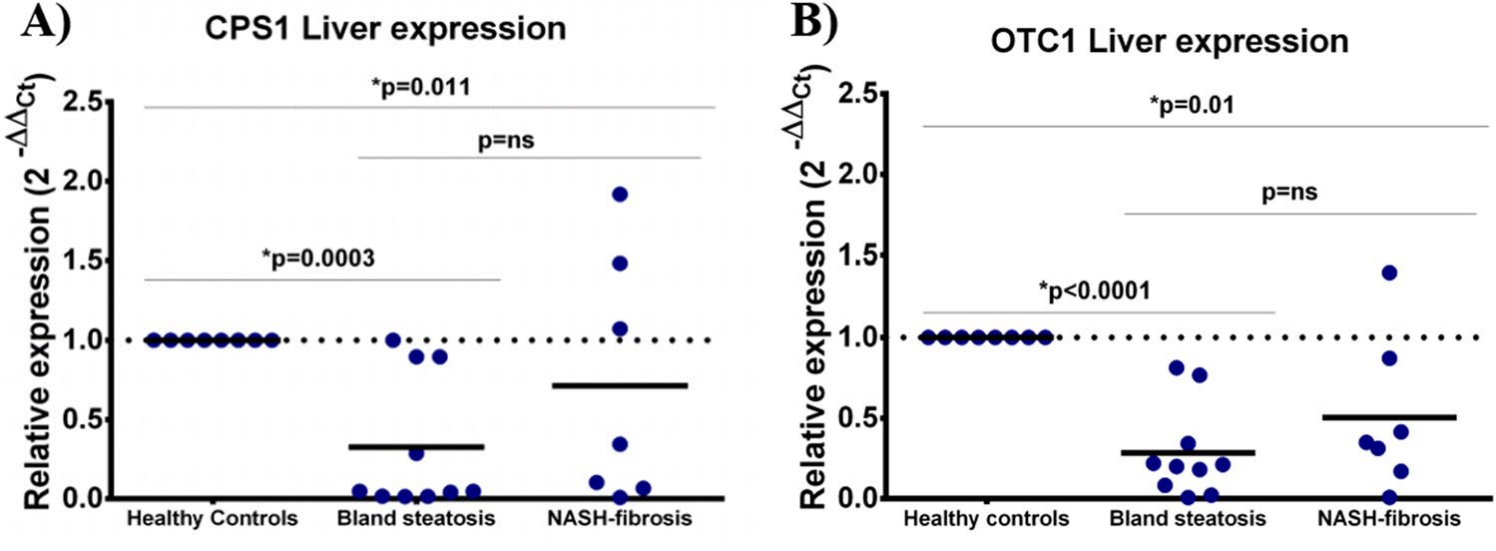
Relative expression results from CPS1 (**A**) and OTC1 (**B**) in liver biopsies from healthy controls, bland steatosis and NASH-fibrosis patients. CPS1: carbamoyl phosphate synthetase-1; OTC1: ornithine transcarbamylase.

Regarding the gene expression, out of the five enzymes analysed, the two mitochondrial-located enzymes showed a significant downregulation in bland steatosis in comparison with healthy controls: CPS1 was 0.32 fold in steatosis [CI95% 0.02–0.63] *vs.* healthy controls (onefold, [CI95% 0.75–1.32]) (*p* = 0.0003), but not statistically significant in patients with NASH-fibrosis when compared to bland steatosis (*p* = ns) (Fig. 1A).

Additionally, there was a downregulation in OTC-1 in bland steatosis (0.28 fold [CI95% 0.08–0.48]) *vs.* healthy controls (onefold [CI95% 0.84–1.18]) (*p* < 0.0001), as well as in NASH-fibrosis patients when also compared to healthy individuals (0.50 fold [CI95% 0.06–0.94]) (*p* = 0.01) (Fig. 1B). No statistical differences were found between both bland steatosis and NASH-fibrosis patients (*p* = ns). CPS1 expression by RNA-seq was found to be diminished in both bland steatosis (0.81 fold; FDR: 0.156) and NASH-fibrosis (0.78 fold; FDR: 0.052) when compared to healthy controls, but only reached statistical significance in the latter (Supplementary Fig. 1A). Further, OTC1 was found to be increased in NASH-fibrosis *versus* healthy controls (0.31 fold, FDR: 0.004) (Supplementary Fig. 1B) Further, in the second subset of patients, CPS1 expression by RNA-seq was found to be diminished in NASH patients without fibrosis when compared to bland steatosis (0.44 fold; FDR: 0.0005) and healthy controls (0.44 fold; FDR: 0.0003) (Supplementary Fig. 1C). Finally, no differences in OTC1 expression were found when comparing these patients (Supplementary Fig. 1D).

**Figure 2:**
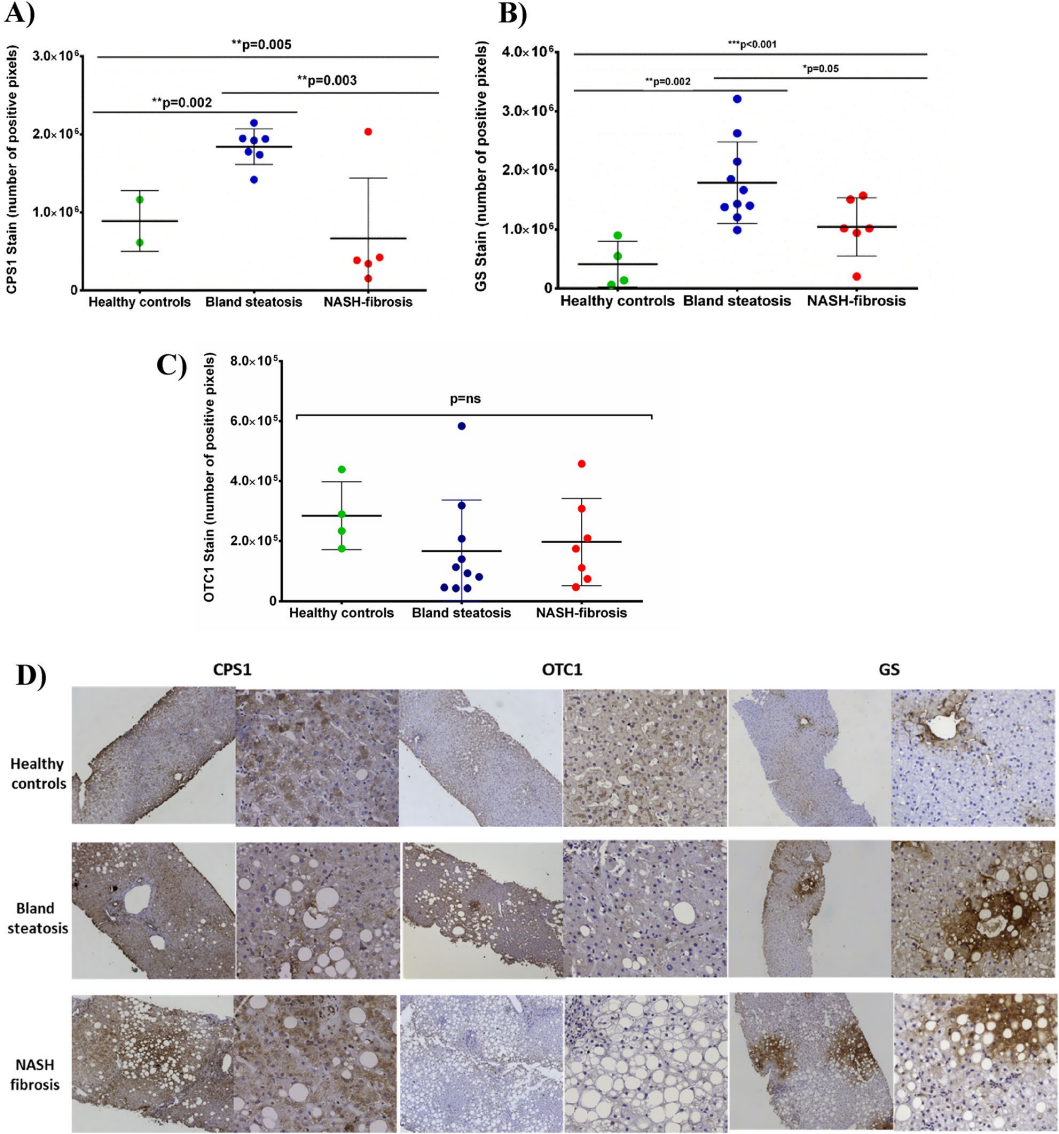
CPS1 (**A**), GS (**B**) and OTC1 (**C**) staining according to different NAFLD stages. (**D**) represents immunohistochemistry findings in human liver biopsies. CPS1: carbamoyl phosphate synthetase-1; GS**:** glutamine synthetase; OTC1: ornithine transcarbamylase.

On the other hand, in the immunohistochemistry staining, CPS1 was also found to be downregulated in NASH-fibrosis patients *versus* bland steatosis (6.6 × 10^5^ ± 7.7 × 10^5^ *vs*. 1.8 × 10^6^ ± 2.2 × 10^5^ stained pixels, *p* = 0.003) (Fig. 2A). Further, CPS1 was found to be increased in bland steatosis patients versus healthy controls (1.8 × 10^6^ ± 2.2 × 10^5^ *vs.* 8.8 × 10^5^ ± 3.8 × 10^5^ stained pixels, *p* = 0.002). The qualitative scale showed that 60% (3/5) of patients with NASH-fibrosis were low positive for CPS1 and none of the simple steatosis patients were found to be low positive (*p* = 0.01).

Further, OTC1 levels were not found to be statistically significant in patients with NASH *versus* steatosis simple patients or *versus* healthy controls (1.9 × 10^5^ ± 1.4 × 10^4^ *vs.* 1.6 × 10^5^ ± 1.7 × 10^5^ or *vs*. 2.8 × 10^5^ ± 1.1 × 10^5^ positive pixels, *p* = ns) (Fig. 2C).

Finally, glutamine synthetase (GS) was found to be upregulated in simple steatosis *versus* healthy controls (1.7 × 10^6^ ± 6.9 × 10^5^ *vs.* 4.1 × 10^5^ ± 3.8 × 10^5^ positive pixels; *p* = 0.002). These levels were found to be downregulated when compared NASH-fibrosis patients *versus* steatosis simple (1.0 × 10^6^ ± 4.9 × 10^5^; *p* = 0.05) (Fig. 2B). The qualitative scale showed that 90% (9/10) of patients with simple steatosis were categorized as highly positive staining and 50% (3/6) of the NASH patients (*p* = ns), and none of the healthy controls were found to be highly positive (*p* = 0.001 when compared with simple steatosis patients). All the immunohistochemistry results are shown in Fig. 2D.

### Findings in rs10478941 variant from CPS1

Main features of this cohort, composed by 382 biopsy-proven NAFLD patients according to genotypes distribution are provided in Supplementary Table 1. Liver fibrosis was distributed as follows: F0 58.4% (223/382), F1 22.3% (85/382), F2 7.9% (30/382), F3 8.6% (33/382) and cirrhosis 2.9% (11/382). In this cohort, 8.6% (33/382) of patients bore AA genotype from CPS1 rs10478941 SNP, 37.2% (142/382) AC genotype and 54.2% (207/382) CC genotype. None of the patients exhibiting AA genotype suffered from liver cirrhosis, and just 12.1% (4/33) showed significant fibrosis in liver biopsy (Supplementary Fig. 1). Further, 64.2% (113/175) of patients bearing A-allele from CPS1 variant did not display hepatic fibrosis in liver biopsy *vs*. 53.4% (110/207) of patients without A-allele and without fibrosis (*p* = 0.03). Nevertheless, there was no association between A-allele and NASH when compared to simple steatosis patients (57.3% (71/124) *vs.* 52.2% (117/224), *p* = ns).

The following variables were found to be associated with liver fibrosis presence in univariate analysis: age (*p* = 0.005), BMI higher than 30 kg/m^2^ (*p* = 0.001), type 2 diabetes mellitus (*p* ≤ *0.001*), AST (*p* ≤ *0.001*), ALT (*p* ≤ *0.001*), GGT (*p* = 0.023), triglycerides (*p* = 0.008) and A-allele from rs1047891 SNP (*p* = 0.036). In multivariate analysis, age (O.R. 1.02; 95% CI 1.02–1.05; *p* = 0.003), BMI > 30 kg/m^2^ (O.R. 2.11; 95% CI 1.31–3.41; *p* = 0.002), T2DM (O.R. 2.66; 95% CI 1.39–5.09; *p* = 0.003), ALT (O.R. 1.01; 95%CI 1.01–1.03, *p* ≤ *0.001*) and A-allele from CPS1 (O.R. 0.62 (95% CI 0.39–0.99; *p* = 0.047) were independently associated with presence of liver fibrosis (Table 2).

**Table 2:** Multivariate analysis of factors independently associated with liver fibrosis.

Variable	Univariate analysis			Multivariate analysis	
	Non-fibrotic (n = 224)	Fibrotic (n = 159)	*p*-value	OR [95%CI]	*p*-value
Sex distribution (Males, %)	43.8%	39.6%	0.413		
BMI > 30 kg/m^2^	31.7%	48.5%	*0.001*	2.11[1.31–3.41]	*0.002*
Age (years)	44.8 ± 12.9	48.7 ± 13.1	*0.005*	1.02[1.01–1.05]	*0.003*
T2DM (yes)	9%	28.3%	≤ *0.001*	2.66[1.39–5.09]	*0.003*
AST (IU/mL)	29.7 ± 21.7	39.3 ± 24.1	≤ *0.001*		
ALT (IU/mL)	41 + 33.1	63.1 ± 44	≤ *0.001*	1.01[1.01–1.03]	≤ *0.001*
GGT (IU/mL)	70.5 ± 79.4	90.5 ± 85.3	*0.023*		
Triglycerides (mg/dL)	123.4 ± 63	157.2 ± 92.5	*0.008*		
Total cholesterol (mg/dL)	190.8 ± 45.6	191.4 ± 43.1	0.941		
Albumin (mg/dL)	4279 ± 329.4	4424.1 ± 463.5	0.076		
CPS1 A-allele (AA/AC vs CC)	50.4% (113/224)	39.6% (63/159)	*0.036*	0.62 [0.39–0.99]	*0.047*

*BMI* body mass index, *T2DM* type 2 diabetes mellitus, *AST* aspartate aminotransferase, *ALT* alanine aminotransferase, *GGT* gamma glutamil transferase, *CPS1* carbamoyl phosphate synthetase-1. Significant values are in Italics.

Finally, ammonia was stained in liver sections of twenty-five NAFLD biopsy-proven patients. Main features of this cohort are described in Supplementary Table 1. Regarding ammonia stain, AA-carriers from rs1047891 SNP showed a significantly reduced levels of hepatic ammonia than AC or CC-carriers (6.7 ± 2.5 *vs*. 25.4 ± 7.10 *vs*. 20.8 ± 5.5 percentage of area stained; *p* = 0.002 and *p* = 0.027 respectively). No significant differences between AC and CC-carriers were found (*p* = ns) (Supplementary Fig. 1).

### UCEs expression in animal models

#### *CDA-HFD model* (Choline-deficient high-fat amino-acid controlled diet with 0.1% added methionine)

**Figure 3:**
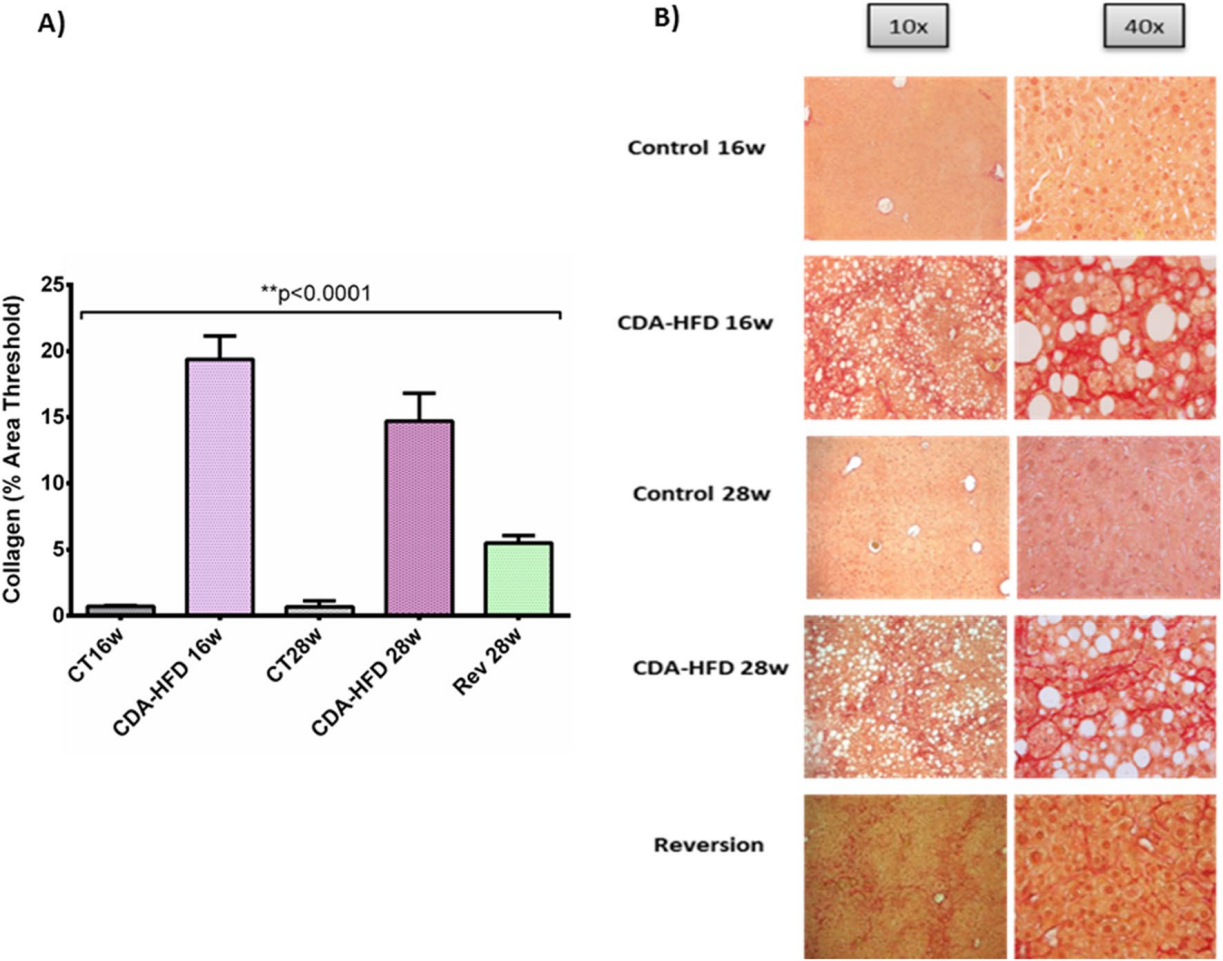
Liver fibrosis in CDA-HFD animals. (**A**) represents the percentage of collagen from the CDA-HFD animals and (**B**) shows the staining. CT: Control; CDA-HFD: choline deficient high-fat diet; Rev: reversion.

**Figure 4:**
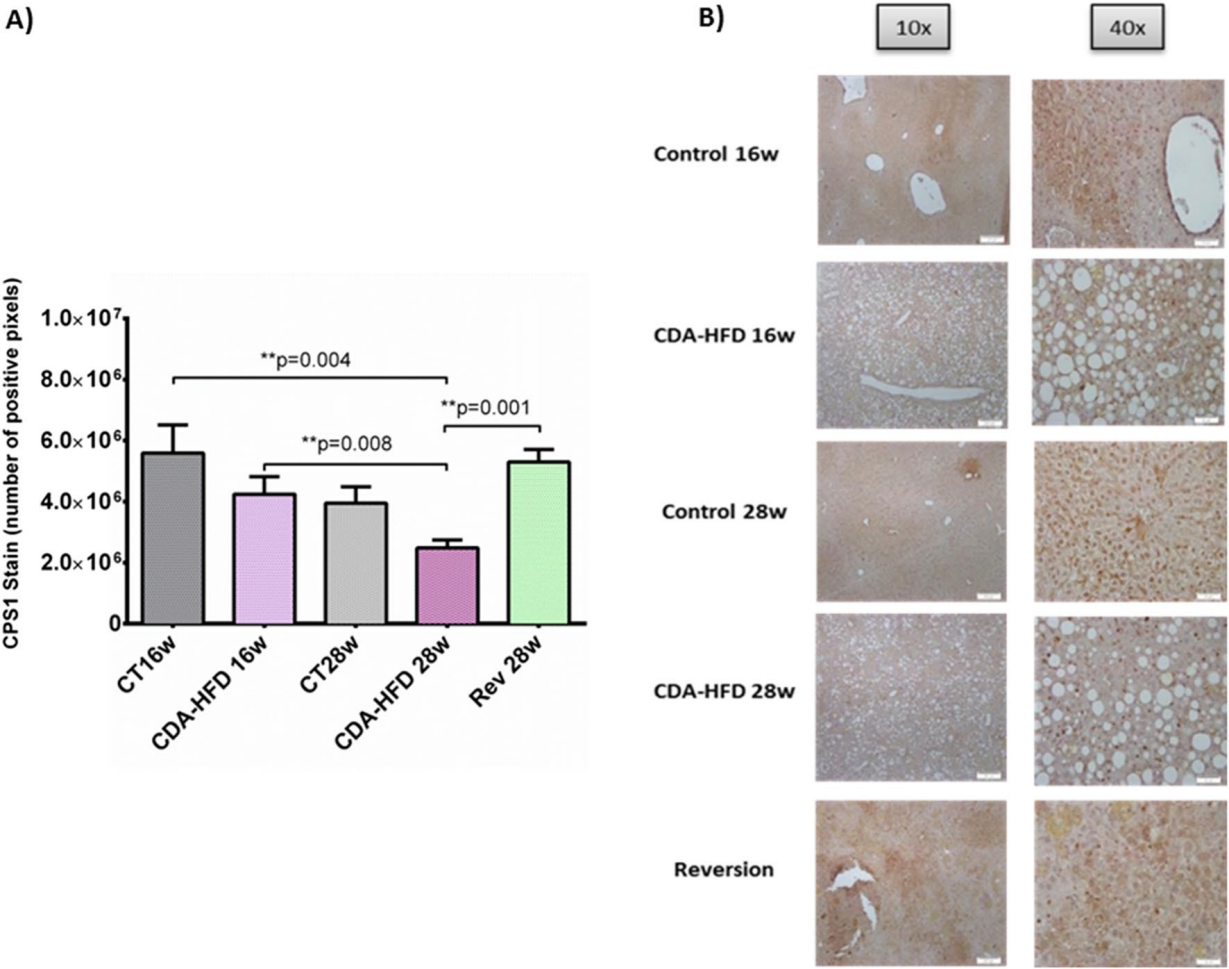
CPS1 findings in CDA-HFD animals. (**A**) represents the number of positive pixels quantified by ImageJ and (**B**) shows the staining. CPS1: carbamoyl phosphate synthetase-1; CT: Control; CDA-HFD: choline deficient high-fat diet; Rev: reversion.

First, liver fibrosis was found to be significantly higher in CDA-HFD groups when compared with controls, at both 16w (19.4 ± 1.76% *vs*. 0.71 ± 0.05% area threshold, *p* = 0.005) and 28w (14.70 ± 2.11% *vs*. 0.66 ± 0.45% area threshold, *p* = 0.023). On the other hand, after 4 weeks with reversion diet, liver fibrosis was found to be diminished but still significant when compared to the CDA-HFD 28w group (5.40 ± 0.52 *vs*. 14.70 ± 2.11 area threshold; *p* = 0.003) (Fig. 3). A significant decrease in CPS1 expression evaluated by IHC was observed after 28 weeks of treatment when compared to control group at 16 weeks (2.5 × 10^6^ ± 2.6 × 10^5^ *vs*. 5.59 × 10^6^ ± 9.2 × 10^5^ positive pixels, *p* = 0.004). Further, CPS1 protein expression was found to be decreased when compare both CDA-HFD, 28 and 16 weeks (2.5 × 10^6^ ± 2.6 × 10^5^ *vs*. 4.24 × 10^6^ ± 5.7 × 10^5^ positive pixels; p = 0.008). Finally, in the reversion group, it was observed that just after 4 weeks of administration of control diet, CPS1 expression was found to be reverted up to control levels (5.3 × 10^6^ ± 4.1 × 10^5^ positive pixels, *p* = ns) (Fig. 4).

#### LDLr knockout animals (knockout for LDL receptor animals)

**Figure 5:**
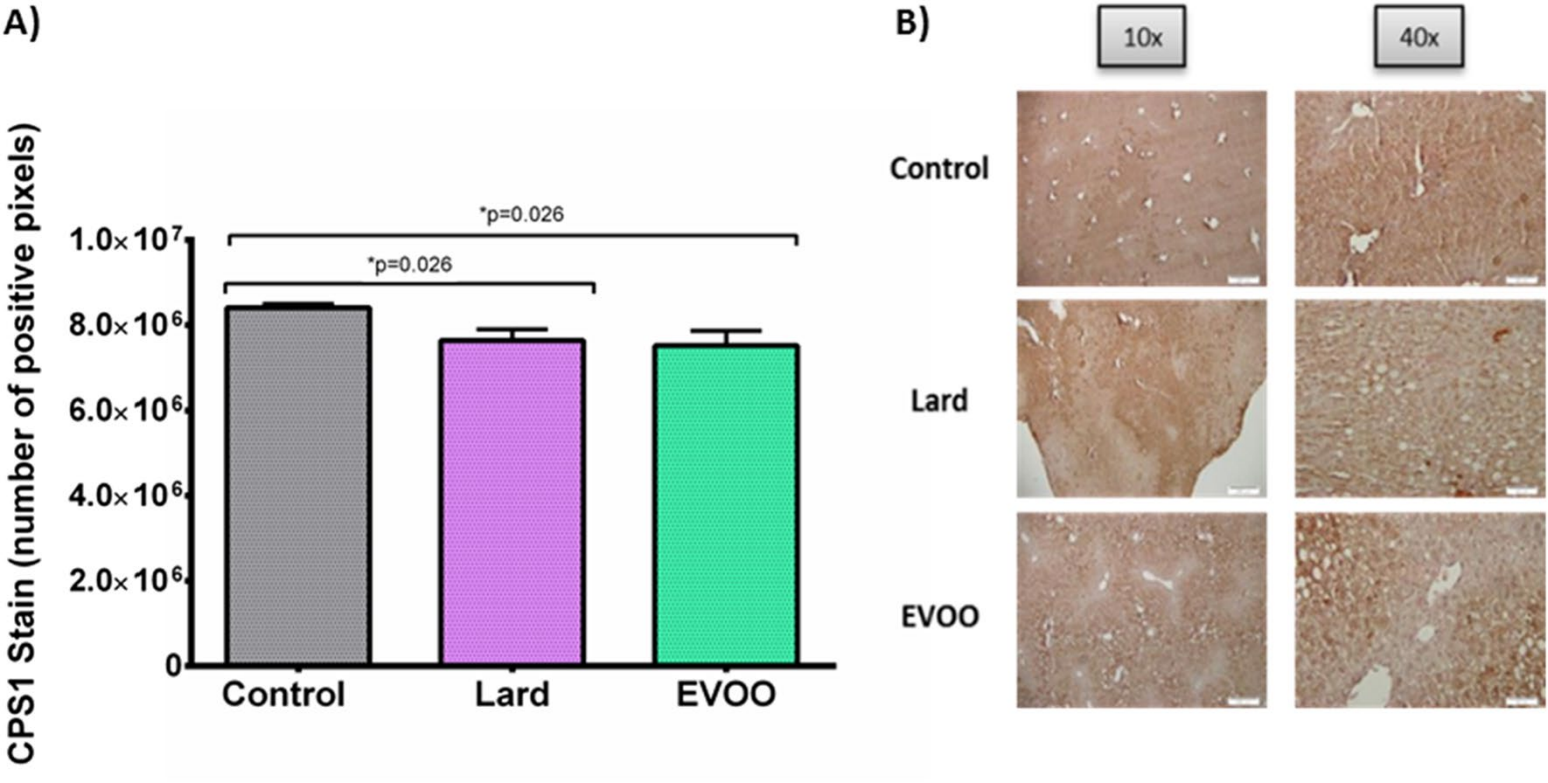
CPS1 findings in LDLr −/− animals. (**A**) represents the number of positive pixels quantified by ImageJ and (**B**) shows the staining. CPS1: carbamoyl phosphate synthetase-1; EVOO: extra virgin olive oil.

Hepatic fibrosis was found to be similar in all groups analysed (0.94 ± 0.05% area threshold for Lard and 1.91 ± 0.28% area threshold for EVOO group) when compared with controls (1.06 ± 0.17% area threshold) (*p* = ns) (Supplementary Fig. 4). After 24 weeks of diet, it was observed a decrease in CPS1 expression when compared to control group, regardless of the type of HFD, lard (8.41 × 10^6^ ± 8.91 × 10^4^ *vs*. 7.64 × 10^6^ ± 2.6 × 10^5^ stained pixels; *p* = 0.026) or extra virgin olive oil (7.51 × 10^6^ ± 3.45 × 10^5^ stained pixels, *p* = 0.026). There were no statistical differences among both groups (*p* = ns) (Fig. 5).

## Discussion

This study has demonstrated that both liver fibrosis and steatosis are associated with a reduction in gene and protein expression patterns of mitochondrial urea cycle enzymes. Our results show that CPS1, the first step limiting enzyme of the cycle, diminishes in parallel with the increase of the liver injury after a deleterious diet, such as CDA-HFD, and this CPS1 impairment could be reversed when the main trigger disappears. We have also confirmed an upregulation in GS protein expression, an ammonia scavenger, which might exert a compensatory effect to avoid ammonia gathering when urea cycle enzymes are impaired. Moreover, we have revealed the potential role of a functional variant previously linked with abnormal ammonia rates in hepatic fibrosis development, leading to a more aggressive phenotype and increased hepatic ammonia levels. This study might open novel hypotheses positioning CPS1 as a novel therapeutic target.

The liver is the main organ responsible for removing nitrogen from blood to synthetize urea in order to be excreted from the body. When ammonia is not successfully cleared from the organism, hyperammonemia could promote different outcomes, from the liver to the brain. Ammonia seems to promote NAFLD progression mainly through the activation of hepatic stellate cells^7^. NASH is considered the progressive entity of NAFLD. Among the intra-organ factors that trigger NASH development it has been described lipotoxicity, innate immune response, apoptosis and cell death, endoplasmic reticulum stress and mitochondrial dysfunction^13,14^. Moreover, ammonia might generate an increased yield of reactive oxygen species that could eventually foster liver injury by mediating inflammatory processes, cell death and fibrosis, hallmarks of NASH^15^. Our findings continue along the same path, due to the main enzymes that found to be dysregulated are both located in this cellular compartment.

In a recent study where authors created a mathematical model to determine changes in ammonia levels from variable dietary protein intake and varying liver enzyme activity, it was reported that CPS1 activity had by far the strongest effect on blood ammonia levels of any other enzyme of the model. They demonstrated that 50% of decreased CPS1 activity led to a more of doubling in blood ammonia levels, as well as the decrease of protein intake may decrease blood ammonia levels minimizing the risk of developing hepatic encephalopathy^16^. Further, it was recently demonstrated that using a metabolite panel reflecting defects in ammonia recycling, urea cycle and amino acid metabolism could detect patients at risk of hepatocellular carcinoma^17^.

It has been previously proven in rats fed diet-inducing NAFLD that steatohepatitis features were associated with a reduction in both CPS1 and OTC expression^5^. The CDA-HFD model has been reported as a good model to recapitulate fibrotic NASH features, due to the hepatic fibrosis is robust and poorly reversible^18^. The urea cycle enzyme and the in vivo capacity for urea-nitrogen synthesis were previously evaluated in a rat model of diet-induced NASH, concluding that advanced NASH resulted in a functional reduction of the capacity of ureagenesis^5^. A further study has revealed the role of hepatic steatosis in hyperammonaemia, and its association with liver fibrosis progression, that may be prevented with the use of ornithine phenylacetate, an ammonia scavenger^7^. Our findings are in alignment with this, due to CPS1, the rate-limiting first step enzyme of urea cycle reduces its levels in parallel with the increase of the hepatic fibrosis. When the injury is reverted, CPS1 levels are restored to normality. Nevertheless, CPS1 protein was found to be increased in steatosis simple patients, probably as a compensatory mechanism to restore hepatic homeostasis during the first stages of liver injury.

Ammonia is a key molecule that might be involved in NAFLD progression, more specifically in liver fibrosis development through hepatic stellate cells activation. That is the main reason to evaluate the impact of a variant located in CPS1 in fibrosis progression. A mechanistic explanation for the involvement of this SNP in the urea cycle activity was previously given. This missense SNP, rs104781, is a C to A nucleotide transversion located in the coding part of CPS1 (exon 36), and results in a substitution of asparagine for threonine in the N-acetylglutamate-binding domain, an important cofactor^19^. The C-encoded form is considered the evolutionary preserved form, and the A-encoded form appears to be a gain of function mutation^19^. This SNP, located in CPS1 gene, was previously linked to functional significances by disturbing the downstream disposal of urea cycle intermediates. It has been reported that the A-allele of rs1047891 might contribute to the availability of precursors for nitric oxide synthesis by influencing nitric oxide production and vascular smooth muscle reactivity^19^, lower platelet count^20^, neonatal pulmonary hypertension^10^, altered lipid profile^10^ and necrotizing enterocolitis susceptibility^21^. Hyperammonaemia occurs during valproic acid treatment (VPA), due to within the hepatic mitochondrial matrix, VPA inhibits CPS1directly and indirectly through the suppression of N-acetyl glutamate and mediating the increase of mitochondrial pyruvate, an inhibitor of CPS1^22^.

Among the limitations of this study there is the lack of ammonia measurements in plasma of these patients. This is mainly due to the challenge in consistency of ammonia assays due to its accuracy is determined by sample timing, condition, handling, storage and assay itself, that may cause pseudo-hyperammonaemia^23,24^. Nevertheless, it was described that in biopsy-proven non-cirrhotic NASH, ammonia levels were found to be higher than their age-paired controls, as well as cognitive impairment associated with hyperammonemia and neuroinflammation^25^.

In conclusion, our study point out CPS1, the pacesetter enzyme of the urea cycle, as a novel therapeutic target. Then, further studies that show correlation between UCEs liver expression, ammonia plasma levels, behavioural analyses and CPS1 variant are warranted.

## Methods

### Study population

CPS1 variant genotyping was performed within a national and multicentre cross-sectional study including a total of 382 biopsy-proven NAFLD patients. Exclusion criteria were: significant alcohol intake (≥ 30 g/day in men and ≥ 20 g/day in women), recreational drugs abuse, evidence of viral or autoimmune hepatitis, HIV, drug-induced fatty liver or other metabolic liver diseases (such as hemochromatosis or Wilson’s disease), together with pregnancy and parenteral nutrition. All patients underwent a screening visit including medical history, physical examination, laboratory tests and signed an informed consent to clinical investigations according to the principles embodied in the Declaration of Helsinki of 1975, as revised in 1983. The Institutional Review Board Committee from each participating Hospital approved the study protocol. Study procedures followed agreed with the ethical standards of the responsible committee on human experimentation, and were approved by the human research ethics committee from each Center (CEI Virgen Macarena/Virgen del Rocío University Hospitals, approval reference C.I. 0359-N-15). Clinical and laboratory data were collected at the same time of liver biopsy. Basic anthropometric data included body mass index (BMI) and abdominal perimeter. An overnight (12 h) fasting blood sample was taken at the same time of liver biopsy for routine biochemical analyses that were performed at the central laboratory of each University Hospital, to rule out occult diseases. Samples were centrifuged 10 min at 3500 revolutions per minute (rpm) right after obtained, aliquoted and immediately stored at −80 °C until assayed. Analyses included transaminases (ALT, AST and GGT), alkaline phosphatase, glucose, insulin, total cholesterol, high-density lipoprotein cholesterol (HDL-c), low-density lipoprotein cholesterol (LDL-c), total bilirubin, albumin, triglycerides. Comorbidities included the presence of type 2 diabetes mellitus, arterial hypertension and dyslipidaemia. Concomitant treatments at the time of biopsy were recorded.

Percutaneous liver biopsies were performed under local anaesthesia and ultrasound guidance. Liver specimens were obtained, after an overnight fast, by "tru-cut" needle (sample length/diameter = 20/1.2 mm) using a biopsy gun and, at least, one sample per patient was extracted. Biopsy samples were fixed in formalin and embedded in paraffin blocks. A fraction was immediately shock-frozen and stored at -80ºC. Specimens were stained with haematoxylin–eosin and Masson´s trichrome. An experienced pathologist blinded with respect to provenance of the samples and unaware of clinical data, assessed the samples using haematoxylin–eosin, reticulin and Masson´s trichrome stains to determine the grading and staging assignments according to NAFLD Activity Score (NAS) and fibrosis stage by Kleiner Score. NAS Score provides an overall score that comprises the degree of steatosis (score 0–3), lobular inflammation (score 0–3) and hepatocyte ballooning (score 0–2). Fibrosis was staged from 0 to 4: stage 0 = no fibrosis, 1 = perisinusoidal or periportal fibrosis, 2 = perisinusoidal and portal/periportal fibrosis, 3 = bridging fibrosis and 4 = cirrhosis. A further 2-level scale of fibrosis was applied: mild (F0-F1) and significant (F2-F3-F4) fibrosis.

Snap-frozen and paraffin-embedded liver tissue was available for both gene expression and immunohistochemistry (IHC) purposes from 17 NAFLD biopsy-proven patients. To avoid biases, patients were selected as bland steatosis (n = 10), defined as steatosis presence, nor steatohepatitis or fibrosis, and NASH with fibrosis (n = 7). Seven healthy controls were also included for gene expression analyses.

### Preclinical models

#### CDA-HFD mice (Choline-deficient high-fat amino-acid controlled diet with 0.1% added methionine)

Five-week old male C57BL6J mice were purchased from Charles River (Cedex, France) and used throughout the study. Mice were bred and maintained in the central animal house. All animal care and experimental protocols were performed in accordance with the guidelines for the care and use of laboratory animals, as well as the European Community Policy for Experimental Animal Studies, in accordance with ARRIVE guidelines. The study was approved by the Institutional Animal Care Committee of CABIMER (permission numbers 06-10-14-138 and 19/02/2016/023). Mice were raised in a temperature-controlled room of 23 °C with constant humidity and maintained on a 12/12-h light/dark cycle. After one week of acclimatization, the mice were fed with the experimental diets. All mice were allowed ad libitum access to the test diets and water throughout the entire research period. Fresh water and a fixed amount of feed were provided three times per week. Body weights were recorded once per week throughout the study period using a calibrated scale, by transferring the mice to a clean empty weighing cage.

Twenty mice were randomly allocated into five groups;

1. Control group, euthanised at 16 weeks (n = 4).
2. Control group, euthanised at 28 weeks (n = 4).
3. CDA-HFD, euthanised at 16 weeks (n = 4).
4. CDA-HFD, euthanised at 28 weeks (n = 4).
5. Reversion group, that followed the CDA-HFD for 24 weeks and then changed to control diet for another 4 weeks (n = 4).

The control group was fed with standard chow, which consisted of a low-fat diet: 13% kcal from fat, 20% kcal from protein and 67% kcal from carbohydrate (2014 Tekland Global Rodent Maintenance Diet, Harlan, Spain); whereas the CDA-HFD group received a high fat diet, deficient in choline and supplemented with 0.1% methionine: 60 kcal% from fat, 18% kcal from protein and 21% kcal from carbohydrates (L-amino acid diet; A06071302, Research Diets Inc, New Brunswick, NJ, USA).

#### LDLr −/− mice (knockout for LDL receptor mice)

Female LDL-receptor knockout Leiden (The Jackson Laboratory, Bar Harbor, Maine, USA) 12-weeks old mice were fed two different high fat diets for 24 weeks and compared with mice fed the standard low-fat diet (LFD).

1. Control diet (n = 2): standard low-fat diet, 13% kcal from fat, 20% kcal from protein and 67% kcal from carbohydrate.
2. Lard (n = 2): lard-HFD, 48% kcal from fat, 12% kcal from protein and 40% kcal from carbohydrate.
3. Extra Virgin Olive Oil (EVOO) enriched in phenolic compounds (n = 2): with an increased and natural content of phenolic compounds (79 mg/kg), 48% kcal from fat, 12% kcal from protein and 40% kcal from carbohydrate.

### UCEs expression: protein and gene hepatic evaluation

Total RNA from frozen liver biopsies was isolated by using mirVana miRNA isolation kit (Life technologies) according to the manufacturers´ protocol. RNA Integrity number (RIN) was measured by electrophoresis (Agilent Technologies, Inc.) to ensure the quality of samples. All tissues showed a RIN higher than 7.5. cDNA synthesis was performed from 500 ng of starting material. qRT-PCR reactions were carried out in triplicate by using 7500 Fast Real-Time PCR System. The five main enzymes of the urea cycle were evaluated: Carbamoyl phosphate synthetase-1 **(**CPS1; Hs_CPS1_1_SG QuantiTect, QT00026705), Ornithine transcarbamylase (OTC; Hs_OTC_1_SG QuantiTect, QT00019509); Argininosuccinate Synthetase (ASS; Hs_ASS1_1_SG QuantiTect; QT00036456); Arginase-1 (ARG; Hs_ARG1_1_SG QuantiTect; QT00068446) and Argininosuccinate lyase (ASL, BioRad; qHsaCID0036503) by using SYBR Green technology. RNA normalization was performed by amplification of RNA 18S as an endogenous control. The 2-ΔΔCT method was used for the analysis of the relative gene expression and results were expressed as fold change.

CPS1 behaviour was further analysed in a second cohort of NAFLD patients (n = 50) in whom differential expression by RNA-seq analysis was recently performed (dataset GSE130970)^26^. All patients included in this study had a biopsy-proven NAFLD, except control subjects that were either donors for living donor transplant or had a prior history of ALT fluctuations that was evaluated with a liver biopsy. Groups compared in this study were classified using the NAS Score and the Kleiner Score as follows: (i) Control (n = 7): healthy individuals; (ii) Bland Steatosis (n = 31): individuals with steatosis without significant fibrosis (F0-F1) and a NAS score ≤ 5 and (iii) NASH-fibrosis: (n = 12) individuals with significant fibrosis (F2-F4) and NAS score ≥ 5. Gene counts were estimated using Tximport v0.99.8 and differential expression analysis was performed with edgeR filtering out genes that had less than 5 counts per million (CPM) in more than 5 samples (minimal sampling group). Finally, CPS1 behaviour was also analysed in a third cohort of NAFLD patients (n = 45) in whom differential expression by RNA-seq analysis was recently performed (dataset GSE126848)^27^. This cohort included 14 healthy normal-weight patients, 15 patients with simple steatosis and 16 NASH patients without liver fibrosis.

Further, liver immunohistochemistry was performed following standard procedures, and liver sections were incubated for 1 h with either Glutamine synthetase (GeneTex, GTX109121, dilution 1:250), OTC (Abcam, ab55914, dilution 1:150) and CPS1 (Santa Cruz, sc-376190, dilution 1:150) antibodies. Images were captured with the Olympus BX-61 direct microscope. Quantification was carried out at 40 × magnification (n = 4 pictures each) with the Open Source Plugin IHC Profiler protocol previously optimized^28^. This plugin offers the pixel-by-pixel quantification, and after calculations the results are expressed as number of positive pixels from the total number of pixels from each image. Further, a 4-level qualitative scale was also obtained according to pixel intensity values: highly positive, positive, low positive and negative.

Finally, ammonia was stained in a total of 25 liver sections from patients bearing different genotypes of rs1047894 variant located in CPS1. Briefly, paraffin-embedded sections were incubated with Nessler´s reagent following the protocol as previously described^29^.

### CPS1 rs1047891 genotyping

DNA was automatically isolated from 400 µL of whole blood by employing Magnapure® Compact equipment (Roche Diagnostics) following manufacturer´s protocol. DNA quantification was performed by NanoDrop™ 2000® (Wilmington, USA) to avoid chemical interferences in the process. CPS1 rs1047891 variant was determined by allelic discrimination using a predesigned Taqman assay (Applied Biosystems, Foster City, CA, EEUU).

### Statistical analyses

Statistical analyses using *t*-tests or ANOVA were carried out for normal distributions, and U-Mann Whitney or Kruskal–Wallis tests were carried out for non-normal variables. Categorical variables were explored by *χ*^2^-analysis, and finally, continuous variables were assessed by Pearson correlation coefficient. Variables showing *p*-values ≤ 0.05 in univariate analysis were entered into backward Wald logistic regression analysis one at the time for genotyping evaluation, to escape from potentially confounding factors and identify factors related to steatohepatitis and liver fibrosis (a significance level of 0.05 was used to eliminate them from the model). Odds ratios (OR) and their 95% confidence intervals (CI) were estimated. The method used for missing data was complete-case analysis since statistical packages exclude individuals with any missing value. For CPS1 rs10478941 variant statistical analyses were performed comparing absence of liver fibrosis (F0) versus presence of fibrosis (F1-F2-F3-F4).

## Supplementary Information

Supplementary Information.

## Abbreviations

NAFLD: Non-alcoholic fatty liver disease
NASH: Non-alcoholic steatohepatitis
CPS1: Carbamoyl phosphate synthetase-1
CDA-HFD: Choline deficient high-fat diet model
LDLr −/−: LDLr knockout model
OTC1: Ornithine transcarbamylase
UCEs: Urea cycle enzymes
GS: Glutamine synthetase
BMI: Body mass index
rpm: Revolutions per minute
HDL-c: High-density lipoprotein cholesterol
LDL-c: Low-density lipoprotein cholesterol
NAS Score: NAFLD Activity Score
IHC: Immunohistochemistry
EVOO: Extra virgin olive oil
RIN: RNA Integrity number
ASS: Argininosuccinate synthetase
ARG: Arginase-1
