# Supplementary material for: Liver injury in non-alcoholic fatty liver disease is associated with urea cycle enzyme dysregulation
Source: Sci Rep. 2022 Mar 1;12:3418. doi: 10.1038/s41598-022-06614-9 (PMC8888708; doi:10.1038/s41598-022-06614-9)
Supplement: Supplementary file 1 — Supplementary Information. [file 41598_2022_6614_MOESM1_ESM.docx]

**Supplementary material**

**Manuscript category:** Original research.

**Title:** Liver injury in non-alcoholic fatty liver disease is associated with urea cycle enzyme dysregulation

**Running tittle:** UCEs and NAFLD.

**List of authors:**

Rocío Gallego-Durán^1,2,*,**^, Javier Ampuero^1,2,3,*^, Helena Pastor-Ramírez^1,2^, Leticia Álvarez-Amor^4,5^, Jose Antonio del Campo^6^, Douglas Maya-Miles^1,2^ , Rocío Montero-Vallejo^1,2^ , Antonio Cárdenas-García^4,5^, Mª Jesús Pareja^7^, Sheila Gato-Zambrano^1,2^, Raquel Millán^1,2^, María del Carmen Rico^1,2^, , Amparo Luque-Sierra ^4,5^, Antonio Gil-Gómez^1,2^, Ángela Rojas^1,2^, Rocío Muñoz-Hernández^1,2^ , María García-Lozano^1,2^, Rocío Aller^8^, Raúl J Andrade^9^, Carmelo García-Monzón^10,2^, Fausto Andreola^11^, Francisco Martín^4,5^, Rajiv Jalan^11,^ Manuel Romero-Gómez^1,2,3,**.^

*These authors contributed equally to this work.

**These authors share co-senior authorship.

**Affiliations:**

1. SeLiver Group, Instituto de Biomedicina de Sevilla/CSIC/Hospital Virgen del Rocío; Sevilla, Spain.
2. Hepatic and Digestive Diseases Networking Biomedical Research Centre (CIBERehd).
3. Digestive Diseases Unit, Hospital Universitario Virgen del Rocío, Sevilla, Spain.
4. Andalusian Center of Molecular Biology and Regenerative Medicine-CABIMER- University Pablo Olavide-University of Seville-CSIC, Sevilla, Spain.
5. Biomedical Research Network on Diabetes and Related Metabolic Diseases-CIBERDEM, Instituto de Salud Carlos III, Madrid, Spain.
6. Digestive Diseases Unit, Hospital Virgen de Valme; Sevilla, Spain.
7. Pathology Unit, Hospital Virgen de Valme; Sevilla, Spain.
8. Digestive Diseases Unit, Hospital de Valladolid; Valladolid, Spain.
9. Unit for the Clinical Management Gastroenterology, Instituto de
   Investigación Biomédica de Málaga-IBIMA, Hospital Universitario Virgen
   de la Victoria, Universidad de Málaga, Málaga, Spain.
10. Liver Research Unit, Hospital Universitario Santa Cristina, Instituto de Investigación Sanitaria Princesa; Madrid, Spain.
11. Liver Failure Group, Institute for Liver and Digestive Health, Royal Free Hospital; London, United Kingdom.

**SUPPLEMENTARY MATERIAL**

**Supplementary Figure 1**. Transcriptomic analyses of CPS1 and OTC1 in the first cohort (A & B) and in the second cohort of patients (C &D).

1. **B)**

**

**



C) D)







**Supplementary Table 1. Clinical and analytical characteristics of the study cohort employed for CPS1 rs1047891 variant genotyping.** This table shows the comparison of clinical characteristics between patients carrying AA, AC or CC genotypes. BMI: Body Mass Index; T2DM: Type 2 Diabetes Mellitus; AST: Aspartate aminotransferase; ALT: Alanine aminotransferase; GGT: gamma glutamyl transferase; NASH: non-alcoholic steatohepatitis.

| **Variable** | **AA-genotype (n=33)** | **AC-genotype (n=142)** | **CC-genotype**  **(n=207)** | **p-value** |
| --- | --- | --- | --- | --- |
| **Sex distribution (Males, %)** | 45.5% | 50.7% | 43% | ns |
| **Age (years)** | 45.7±11.3 | 45.7±13.8 | 47±12.9 | ns |
| **BMI (kg/m^2^)** | 33.6±8.8 | 34.5±9.5 | 34.3±10.2 | ns |
| **T2DM, %** | 6.1% | 15% | 19.9% | ns |
| **AST (IU/mL)** | 34±19 | 31±20 | 35±26 | ns |
| **ALT (IU/mL)** | 51±43 | 49±39 | 51±35 | ns |
| **GGT (IU/mL)** | 70±80 | 75±76 | 83±87 | ns |
| **Glucose (mg/dL)** | 97±19 | 103±30 | 105±33 | ns |
| **Insulin (microUI/mL)** | 16±14 | 14±9 | 13±11 | ns |
| **Triglycerides (mg/dL)** | 157±102 | 120±62 | 144±80 | ns |
| **Total cholesterol (mg/dL)** | 203±45 | 185±41 | 193±47 | ns |
| **Albumin (mg/dL)** | 4352±309 | 4289±329 | 4360±439 | ns |
| **Platelet count, ^10^9^** | 240±53 | 234±70 | 222±68 | ns |
| **NAS Score** | 2.6±1.99 | 1.6±1.5 | 2.1±2 | ns |
| **NASH** | 37.9% | 31.5% | 38.1% | ns |
| **Liver fibrosis (presence)** | 39.4% | 35.2% | 46.4% | ns |

**Supplementary Figure 2.** CPS1 rs1047891 genotypes distribution according to liver fibrosis stages in NAFLD patients. CPS1: carbamoyl phosphate synthetase-1.

**Supplementary Table 2. Clinical and analytical characteristics of the study cohort employed for ammonia staining.** This table shows the comparison of clinical characteristics between patients carrying AA, AC or CC genotypes of **CPS1 rs1047891 variant**. BMI: Body Mass Index; T2DM: Type 2 Diabetes Mellitus; AST: Aspartate aminotransferase; ALT: Alanine aminotransferase; GGT: gamma glutamyl transferase; NASH: non-alcoholic steatohepatitis, CPS1: carbamoyl phosphate synthetase-1. Significant values are in Italics

| **Variable** | **AA-genotype (n=2)** | **AC-genotype (n=13)** | **CC-genotype**  **(n=10)** | **p-value** |
| --- | --- | --- | --- | --- |
| **Sex distribution (Males, %)** | 50%(1/2) | 53.8%(7/13) | 60%(6/10) | p=0.94 |
| **Age (years)** | 65.5±0.7 | 42.4±14.4 | 47.6±13.1 | *p=0.06* |
| **BMI (kg/m^2^)** | 29.0 | 29.6±5.7 | 28.4±4.7 | p=0.87 |
| **T2DM, %** | 50%(1/2) | 15.4% (2/13) | 30% (3/10) | p=0.48 |
| **AST (IU/mL)** | 79.5±70 | 46.4±41.5 | 35±15.4 | p=0.27 |
| **ALT (IU/mL)** | 104.5±82.7 | 53.1±34.3 | 48.7±23.4 | p=0.13 |
| **GGT (IU/mL)** | 82.5±19.1 | 61.6±46.6 | 137.5±144.6 | p=0.21 |
| **Glucose (mg/dL)** | 109±18.4 | 101.2±37.8 | 88.8±16.2 | p=0.53 |
| **Insulin (microUI/mL)** | 17.6±12.2 | 12.1±10.4 | 13.1±5.7 | p=0.72 |
| **Triglycerides (mg/dL)** | 212.5±95.5 | 97.5±53.5 | 107.6±41.1 | *p=0.03* |
| **Total cholesterol (mg/dL)** | 270±70.7 | 180.8±46.8 | 207.8±37.1 | *p=0.04* |
| **NAS Score** | 2.0±1.4 | 2.0±1.7 | 1.7±1.5 | p=0.9 |
| **Fibrosis stage** | 2±1.4 | 0.8±1.2 | 0.9±1.1 | p=0.41 |

**Supplementary Figure 3. Ammonia staining in human liver samples according to different genotypes of CPS1 rs1047891 variant.** A) Percentage of area stained according to CPS1 rs104781 varian; B) Representative Nessler´s staining according to CPS1 rs104781 variant. CPS1: carbamoyl phosphate synthetase-1.

**A)**

**

**

**B)**

**
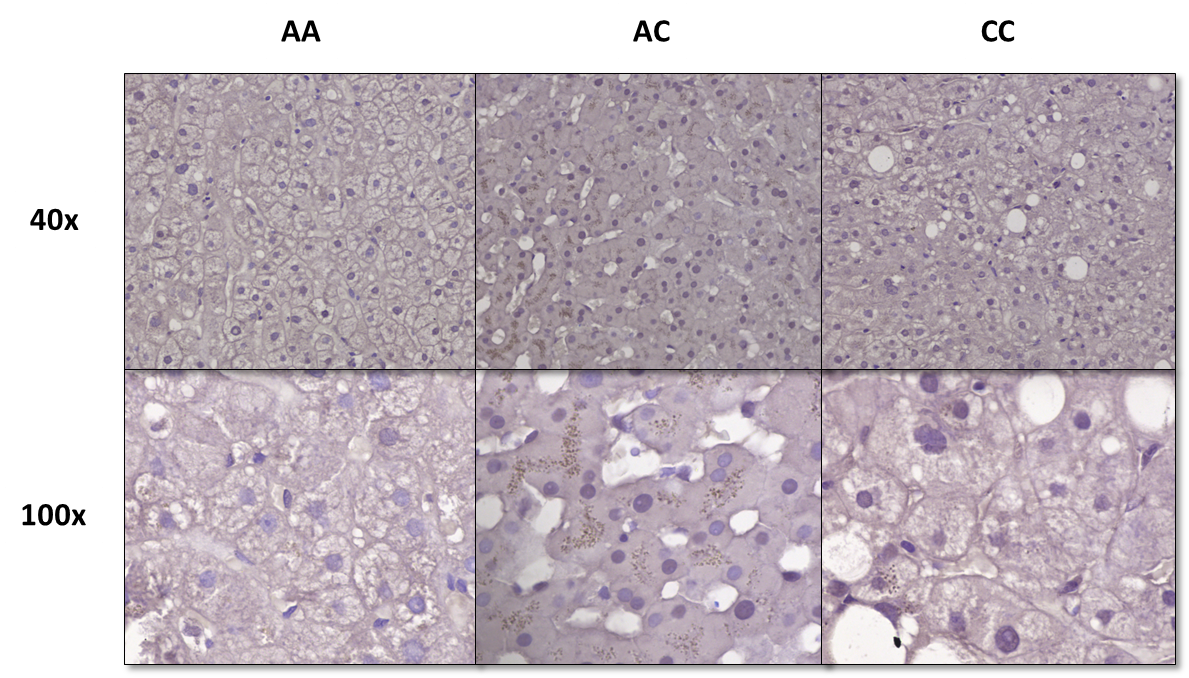
**

**Supplementary Figure 4.** Liver fibrosis in LDLr -/- animals. LDLr **-/-**: LDLr knockout; Ctrl: control.

**


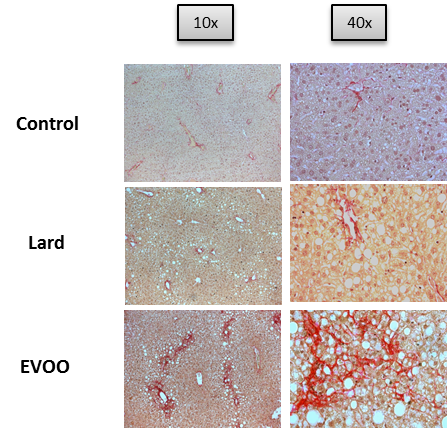
**
